# A case of prominent immunoglobulin G4‐positive lymphadenopathy in response to microscopic lung cancer

**DOI:** 10.1002/rcr2.854

**Published:** 2021-10-03

**Authors:** Motoi Yuzawa, Hiromitsu Ohta, Motoko Nomura, Kentaro Minegishi, Hisashi Oshiro, Yasuhiro Yamaguchi

**Affiliations:** ^1^ Department of Respiratory Medicine, Saitama Medical Center Jichi Medical University Saitama Japan; ^2^ Department of Thoracic Surgery, Saitama Medical Center Jichi Medical University Saitama Japan; ^3^ Department of Pathology, Saitama Medical Center Jichi Medical University Saitama Japan

**Keywords:** EBUS‐TBNA, immunoglobulin G4‐related disease, lung cancer, lymphadenopathy, thoracoscopic lymph node biopsy

## Abstract

Immunoglobulin G4 (IgG4)‐related disease is established as a new clinical entity, characterized by high levels of plasma IgG4 and IgG4‐positive plasma cell infiltration. However, the elevation of plasma IgG4 and infiltration of IgG4‐positive cells have been observed in other diseases, including malignancy. We experienced a case of prominent IgG4‐positive lymphadenopathy, which was diagnosed as a reactive lesion in response to lung cancer. The cancerous lesion was so small in size that it was difficult to reveal the coexisting lung cancer. Surgical lymph node biopsy and endobronchial ultrasound‐guided transbronchial needle aspiration did not reveal lymph node metastasis of cancer. Mediastinal lymph node dissection finally revealed it. After the right upper lobectomy, the patient underwent postoperative chemotherapy and remains cancer‐free after 1 year. Our case suggests that close examination and careful follow‐up are necessary when IgG4‐positive lymphadenopathy is observed.

## Graphical abstract

This article discussed a case in which prominent lymphadenopathy with immunoglobulin G4‐positive cell infiltration was caused in response to microscopic lung cancer. Surgical lymph node biopsy, as well as endobronchial ultrasound‐guided transbronchial needle aspiration, could not reveal coexisting malignant cells in the mediastinal lymph node.

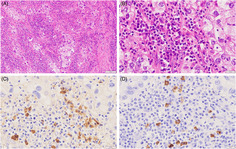

## INTRODUCTION

Immunoglobulin G4 (IgG4) is a minor component that accounts for approximately 4% of IgG; however, its pathological significance has not been revealed. In 2001, a report was published of elevated plasma IgG4 levels and infiltration of IgG4‐positive cells to the lesion in patients with sclerosing cholangitis.^1^ Since then, the pathological significance of IgG4 has been discussed. Similar pathological and clinical features are commonly found in various diseases formerly diagnosed as Mikulicz diseases, such as autoimmune pancreatitis, hypophysitis, autoimmune thyroiditis, interstitial pneumonitis, interstitial nephritis, retroperitoneal fibrosis, inflammatory aortic aneurysm and aortitis. IgG4‐related disease (IgG4‐RD) has been established as a new clinical entity, which manifests as multiple organ diseases characterized by high levels of plasma IgG4 and the infiltration of IgG4‐positive plasma cells. However, it should be emphasized that elevation of plasma IgG4 and infiltration of IgG4‐positive cells are also found in diseases other than IgG4‐RD, including multicentric Castleman disease, malignant lymphoma, cancer, sarcoidosis and collagen vascular diseases.

We experienced a case in which prominent lymphadenopathy with IgG4‐positive cell infiltration occurred in response to lung cancer. The cancerous lesions were so small that the diagnosis of cancer was difficult with endobronchial ultrasound‐guided transbronchial needle aspiration (EBUS‐TBNA) or thoracoscopic lymph node biopsy.

## CASE REPORT

A 54‐year‐old man visited a nearby hospital because of a chronic cough. Chest computed tomography (CT) revealed mediastinal lymphadenopathy and a small subpleural nodule in the right upper lobe. He had a history of smoking 1.5 packs/day for 48 years and worked as a taxi driver. His medical history included childhood asthma, hyperlipidaemia and hypertension.

**FIGURE 1:**
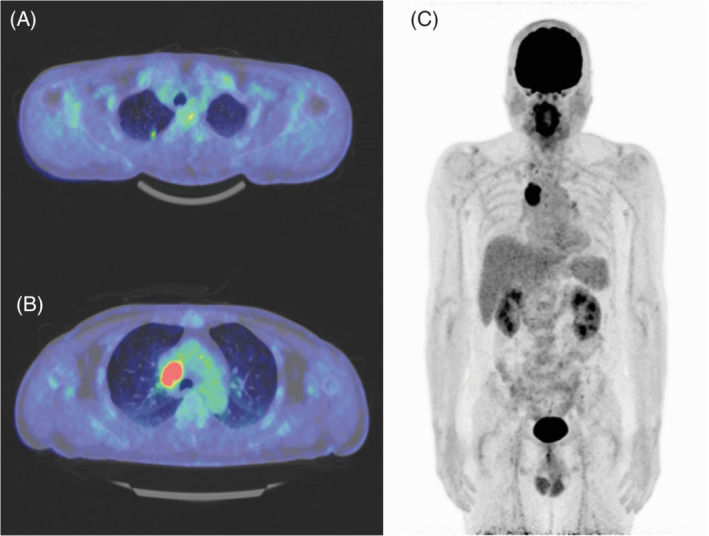
Positron emission tomography‐computed tomography. (A). A nodular shadow was found in the right lung apex, with fluorodeoxyglucose (FDG) accumulation with a maximum standardized uptake value (SUV_max_) of 2.5. A benign nodule, such as inflammatory granuloma, was suspected. (B) The right tracheobronchial lymph node was swollen, with FDG accumulation with an SUV_max_ of 10.7. The density inside the lymph nodes and their margins were smooth, suggesting lymphoproliferative disease. (C) There was no other abnormal uptake of FDG except the above two lesions

He was afebrile, with no swelling of the superficial lymph nodes, and no other abnormalities were observed on physical examination. Laboratory findings revealed a normal blood count, normal hepatic function and normal renal function, except for an increase in C‐reactive protein (8.91 mg/dl). Tumour markers (carcinoembryonic antigen, cytokeratin‐19 fragment, pro‐gastrin‐releasing peptide and soluble interleukin‐2 receptor) were within the normal range. The antinuclear antibody level was not elevated (<40 times). The interferon‐γ release assay yielded negative results. Positron emission tomography (PET)‐CT showed a high accumulation of fluorodeoxyglucose (FDG) in the mediastinal lymph nodes and a low accumulation of FDG in the small nodule in the right upper lobe (Figure 1).

**FIGURE 2:**
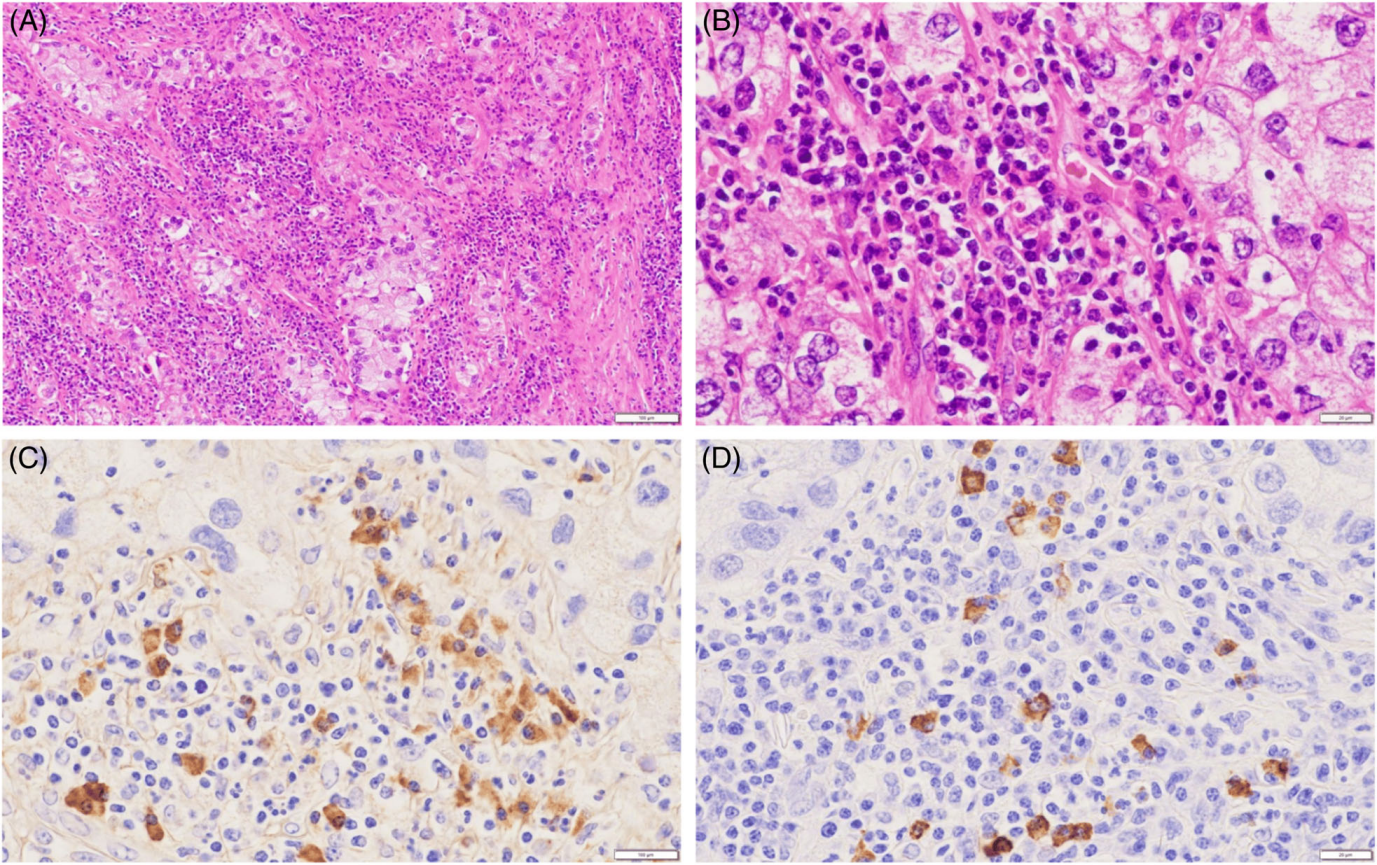
Photomicrographs of pulmonary adenocarcinoma metastasizing into the lymph node. (A) Metastatic adenocarcinoma accompanied by dense fibrous connective tissue and inflammatory cell infiltration (haematoxylin and eosin stain, scale bar = 100 μm). (B) Prominent lymphoplasmacytic infiltration adjacent to adenocarcinoma cell clusters (haematoxylin and eosin stain, scale bar = 20 μm). (C) Immunoglobulin G (IgG)‐positive plasma cells adjacent to adenocarcinoma cell clusters (immunohistochemistry, scale bar = 20 μm). (D) IgG4‐positive plasma cells adjacent to adenocarcinoma cell clusters (immunohistochemistry, scale bar = 20 μm)

Initially, EBUS‐TBNA for mediastinal lymphadenopathy was performed. The specimens implied lymphoproliferative disease; however, their quantity was insufficient for diagnosis. Therefore, a thoracoscopic lymph node biopsy was performed. This revealed no malignant cells in the lymphoid tissue specimen. IgG4‐positive cells were scattered in the lymph nodes and accumulated in the extranodal area, and the IgG4/IgG ratio was approximately 50%, implying IgG4‐RD. Simultaneously, a biopsy of the nodule in the right upper lobe with a thoracoscopy was performed. Although no cancerous cell was found by intraoperative diagnosis, pathological examination of permanent specimens postoperatively revealed adenocarcinoma of 4 mm in size. Right upper lobectomy with lymph node dissection was performed on another day. No malignant lesions other than the nodule already excised were found in the resected lobe. In addition, infiltration of IgG4‐positive cells was not observed. Metastasis of the adenocarcinoma was observed only in a part of the right tracheobronchial lymph node. Dense infiltration of IgG4‐positive plasma cells and fibrosis adjacent to adenocarcinoma cell clusters were observed in the dissected lymph nodes, and the IgG4/IgG ratio was approximately 50%. The number of IgG4‐positive plasma cells per high‐power field was more than 10 (Figure 2). He was diagnosed with stage IIIA (pT1miN2M0) lung adenocarcinoma and IgG4‐positive lymphadenopathy in response to lung cancer. Postoperatively, the IgG4 plasma level was <130 (42.4 mg/dl). The patient underwent postoperative chemotherapy. CT has been performed regularly; no findings suggesting cancer recurrence, such as lymphadenopathy, have been reported for 1 year. The IgG4 plasma level decreased gradually to 17.7 mg/dl.

## DISCUSSION

We concluded that the lymphadenopathy in our case was a reactive lesion of lung cancer for the following reasons. Although the cancer component was minimal, approximately 4 mm in the primary lesion, and metastasis occurred in a limited part of the mediastinal lymph nodes, both were identified at the same time as IgG4‐positive lymphadenopathy. PET/CT revealed no other lesion suspected of IgG4‐RD, and periodic examinations with CT had not shown any relapse lesions of lung cancer or lymphadenopathy 1 year postoperatively.

This case was excluded from IgG4‐RD according to the comprehensive diagnostic criteria for IgG4‐RD revised in 2020 (Table 1).^2^ The comprehensive diagnostic criteria for IgG4‐RD were established by the working group organized by the Ministry of Health, Labour and Welfare of Japan in 2011. It consists of three items: clinical, serological and pathological findings. Although many cases diagnosed by these criteria have been reported worldwide, several problems have been noted in clinical practice. One of them is that lymphadenopathy with infiltration of IgG4‐positive lymphocytes is often found in patients with autoimmune diseases, malignant lymphoma and lymph node metastasis of cancer. The revised diagnostic criteria excluded the diagnosis of IgG4‐RD if lymphadenopathy was the only lesion involved in IgG4, as in our case. About 55%─88% of patients diagnosed with IgG4‐RD have multiple organ involvement. Martinez et al. reported eight patients with IgG4‐positive lymphadenopathy without a clinical sign of IgG4‐RD. None of them had developed IgG4‐RD during follow‐up.^3^

**TABLE 1:** Comprehensive diagnostic criteria for IgG4‐RD revised in 2020. Summary from the original version

(1) Clinical and radiological features: one or more organs with diffuse or localized swelling or a mass or nodule characteristic of IgG4‐RD
(2) Serological diagnosis: serum IgG4 levels greater than 135 mg/dl
(3) Pathological diagnosis: positivity for two of the following three criteria:
(i) Dense lymphocyte and plasma cell infiltration with fibrosis
(ii) The ratio of IgG4‐positive plasma cells/IgG‐positive cells greater than 40% and the number of IgG4‐positive plasma cells greater than 10 per high‐power field
(iii) Typical tissue fibrosis, particularly storiform fibrosis, or obliterative phlebitis

Abbreviation: IgG4‐RD, immunoglobulin G4‐related disease.

*Source*: Umehara et al., 2021.^2^

The relationship between IgG4 and malignant diseases is still unknown. Patients with IgG4‐RD may be prone to malignant tumours. In animal experiments, IgG4 could compete with IgG1 by attenuating the activation of the FCγ receptor. IgG4 may inhibit IgG1‐mediated tumour immunity.^4^

Zen and Nakanuma found three patients with coexisting malignant tumours among 114 patients with IgG4‐RD.^5^ Yamamoto et al. reported 11 patients with coexisting malignant tumours among 106 patients with IgG4‐RD in Japan.^6^ They reported that the prevalence of malignant tumours was 3.5 times higher in these patients than in the healthy population.

Asano et al. also reported that the incidence of malignant disease in patients with IgG4‐RD was higher than that in the control group at 12 years after diagnosing IgG4‐RD.^7^ Malignant diseases were most frequently diagnosed within 1 year. In contrast, according to the report by Hirano et al., there was no significant difference in the incidence of malignant disease between patients with IgG4‐RD and the control population.^8^ These discrepancies could be explained by the fact that Hirano et al. excluded cases in which IgG4‐RD and malignant disease were diagnosed simultaneously to prevent selection bias. Considering that many malignant tumours were diagnosed within 1 year after the diagnosis of IgG4‐RD, there might be some cases in which malignant disease has been overlooked.

Furthermore, Asano et al. pointed out that some cases of IgG4‐RD might occur as a paraneoplastic syndrome. Ito et al. reported that axillary lymphadenopathy with IgG4‐positive plasma cell infiltration occurred during the treatment of lung adenocarcinoma with osimertinib.^9^ Besides, Fujimoto et al. reported that IgG4‐positive plasma cell infiltration was observed around the tumour in non‐small cell lung cancer without clinical findings of IgG4‐RD.^10^

The measurement of the plasma IgG4 level is one of the most useful tools for diagnosing IgG4‐RD and evaluating the disease activity. Xu et al. reported that plasma IgG4 has a very high sensitivity (85%) and specificity (93%) in detecting IgG4‐RD.^11^

Plasma IgG4 level might be helpful to differentiate IgG4‐RD and cancer. Differential diagnosis between IgG4‐RD and cancer is sometimes difficult because IgG4‐RD can form tumour‐like lesions, and desmoplastic tissue around the malignant tumour is similar to the lesion of IgG4‐RD when IgG4‐positive plasma cells are abundant. Plasma IgG4 may be elevated in some patients with cancer, but plasma IgG4 tends to be higher in patients with IgG4‐RD. Oseini et al. reported that 17 (13.5%) of the 126 patients with cholangiocarcinoma had elevated IgG4 (>140 mg/dl) and four (2%) had more than two‐fold elevation (>280 mg/dl), whereas 39 (78.0%) of 50 patients with IgG4‐associated cholangitis had elevated IgG4 (>140 mg/dL) and 25 (50%) had more than two‐fold elevation.^12^ In our case, the plasma IgG4 level had not been measured preoperatively, but it had remained within normal limits after the surgery.

Our case showed marked IgG4‐positive lymphadenopathy in response to lung cancer. Surgical lymph node biopsy, as well as EBUS‐TBNA, could not reveal coexisting malignant lesions. Our case suggests that close examination and careful follow‐up are necessary when IgG4‐positive lesions are observed, especially if the lesion is limited to lymphadenopathy.

## CONFLICT OF INTEREST

None declared.

## AUTHOR CONTRIBUTION

Motoi Yuzawa and Hiromitsu Ohta designed this report and wrote the manuscript. Kentaro Minegishi performed thoracic surgery. Hisashi Oshiro made the pathological diagnosis. Motoi Yuzawa, Hiromistu Ohta and Motoko Nomura participated in the discussion. Yasuhiro Yamaguchi supervised this report. All authors were involved in drafting the manuscript and approved the final version of the manuscript.

## ETHICS STATEMENT

The authors declare that appropriate written informed consent was obtained for publication of this case report and accompanying images.
